# P63 Development and early impact of antimicrobial stewardship (AMS) handbooks for pharmacy technicians across care settings in England

**DOI:** 10.1093/jacamr/dlag102.069

**Published:** 2026-06-26

**Authors:** Alishah Lakha, Naomi Fleming, Jessica Mann, Shazia Nazir, Vikki Connell, Sarah Newsome

**Affiliations:** NHS England, UK; NHS England, UK; NHS England, UK; Leeds Teaching Hospitals NHS Foundation Trust, Leeds, UK; University Hospitals of North Midlands NHS Trust, UK; NHS England, UK

## Abstract

**Objectives:**

To describe the development of AMS Handbooks for pharmacy technicians and evaluate early indicators of impact on knowledge, confidence, and application to practice. The handbooks aim to support consistent engagement in stewardship activities across care settings, particularly for non-AMS specialist roles.

**Methods:**

A cross-collaborative approach was undertaken to develop, review and refine the AMS handbooks. Two dedicated working groups comprising of pharmacy technicians from primary care (including Primary Care Network and General Practice settings) and secondary care (Acute Trust) were established. Each group was responsible for ensuring alignment with real-world roles and practice. Draft versions of the handbooks were circulated for wider review, with feedback obtained from a multidisciplinary group of stakeholders. This included pharmacy professionals across care settings, enabling refinement of content, structure, and usability. Final versions were approved via the East of England AMS regional committees. The primary care and secondary care handbooks were published on a national knowledge-sharing platform between December 2025 and Jan 2026. Early evaluation was conducted using a mixed-methods approach, including analysis of resource engagement metrics (views and downloads) and a rapid evaluation survey targeting pharmacy technicians. The survey captured self-reported changes in knowledge, confidence, and application to practice, alongside free-text responses. Additional qualitative insights were obtained from stakeholder feedback during development and informal discussions within professional forums.

**Results:**

Early uptake indicates strong engagement, with 374 total views/downloads. Survey responses from pharmacy technicians (*n*=10), spanning AMS specialist (6/10) and non-specialist roles (4/10), showed improvements in AMS knowledge, role clarity, and confidence (7/10 for each outcome). All non-specialist respondents reported improvements across these domains. Nine respondents had used or intended to use the handbook, primarily for education, training, or induction. All respondents would recommend the resource. Qualitative results from the survey and wider stakeholder feedback highlighted practicality, relevance for non-specialist roles, and usefulness for induction and protected learning time, with case studies being noted for enhancing engagement.

**Conclusions:**

The co-produced AMS Handbooks demonstrate early evidence of impact in addressing previously identified gaps in AMS education, training, and scope of practice across the pharmacy technician workforce. Engagement data and evaluation findings indicate the resources are particularly valuable for non-specialist and early-career specialist roles, supporting foundational AMS knowledge, role clarity, and confidence to contribute to AMS activities. Their uptake across both primary and secondary care suggests potential to promote greater consistency in AMS capability and delivery. While findings are based on a small sample and should be interpreted as indicative, triangulation of engagement metrics, survey responses, and qualitative feedback strengthens confidence in their early impact. Overall, the results support the value of structured, role-specific resources in operationalizing AMS responsibilities and supporting workforce development. Further evaluation is warranted to assess sustained practice change and contribution to antimicrobial optimization and patient outcomes.

